# Connection and Deformation of Pathological Images via a Macro Image for Comparing Different Modality Images of Brain Tumor

**DOI:** 10.1155/2014/368951

**Published:** 2014-11-19

**Authors:** Takashi Ohnishi, Takuya Tanaka, Yuka Nakamura, Noriaki Hashimoto, Hideaki Haneishi, Jennie Taylor, Matija Snuderl, Yukako Yagi

**Affiliations:** ^1^Center for Frontier Medical Engineering, Chiba University, Chiba 263-8522, Japan; ^2^Department of Medical System Engineering, Chiba University, Chiba 263-8522, Japan; ^3^Graduate School of Engineering, Chiba University, Chiba 263-8522, Japan; ^4^Massachusetts General Hospital, Boston, MA 02114, USA; ^5^New York University Langone Medical Center, New York, NY 10016, USA; ^6^Massachusetts General Hospital Pathology Imaging and Communication Technology (PICT) Center, Boston, MA 02114, USA; ^7^Harvard Medical School, Boston, MA 02215, USA

## Background

Magnetic resonance imaging (MRI) is a preferred modality for diagnosis of brain tumor. However, because infiltrated regions with tumor are often indistinct on the MR image, it is difficult to identify tumor regions exactly. For revealing the relationship between tissue information and MR signal of the tumor, pathological images and MR images at the same regions have to be analyzed. However, it is not easy to make relationship between pathological images and MR images because pathological images are divided and deformed through tissue specimen making. We propose a registration scheme of a set of pathological images and MR image by referring a macro image captured by an optical camera. In the first step, parted pathological images are pieced together and deformed with macro image. In the second step, connected pathological image is registered to MR image. This paper shows connection and deformation methods for the pathological image.

## Method

**Figure 1:**
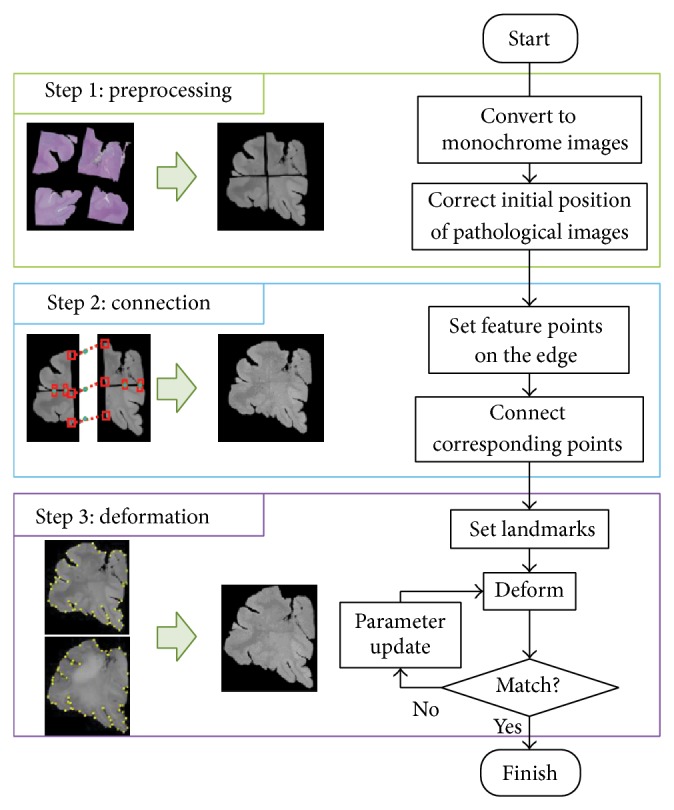
Flow of proposed method.

This paper explains a method for the connection and deformation method of the pathological image with the macro image.  Figure 1 shows the flow of the proposed method. Before the connection step, each image is converted to monochrome with green component for the pathological image or blue component for the macro image, and then initial positions of pathological images are roughly corrected manually. Next, all pathological images at the same slice are connected by use of corresponding feature points. Here, feature points are manually selected from edge region to be connected. While feature points are connected, other regions are deformed by thin plate spline technique. After that, the connected pathological image is deformed referring to the macro image. Deformation step consists of landmark based and intensity based phases. In the landmark based phase, landmarks are chosen from the pathological image and macro image and match them in a similar way to the connection step. Finally, pathological image is deformed using intensity based matching method. Similarity between both images was evaluated by normalized mutual information and maximized by Powell-Brent method.

## Results

**Figure 2:**
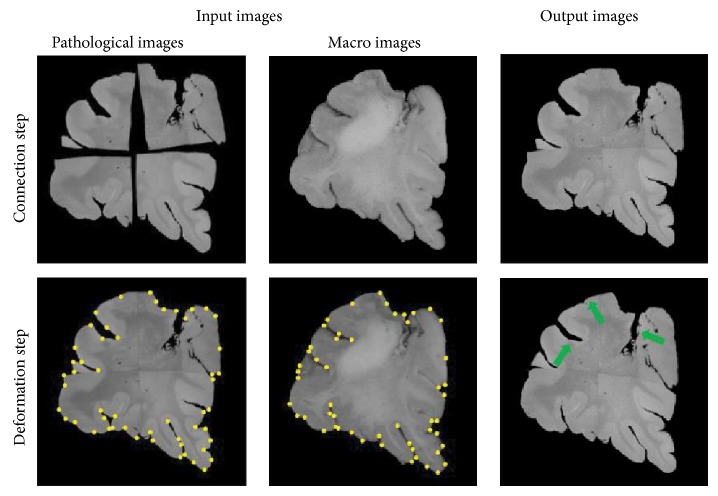
Result of each step.

We applied the proposed method to a dataset taken from a patient of glioblastoma, a kind of the brain tumor.  Figure 2 shows the result of each step. A connected pathological image was successfully obtained from several ones by use of the proposed method. After deformation, the pathological image became more similar to the macro image. Green arrows in Figure 2 represent the remarkable places of successful deformation.

## Conclusion

We developed a connection and deformation method for the pathological images via the macro image. Through a test for dataset of brain tumor, we confirmed the proposed method can rebuild the pathological image before the tissue specimen making. We are currently integrating the proposed method with a registration method using pathological image and MR image.

